# A Fork in the Road: An Unusual Case of Duodenal-Cecal Fistula Due to a Chronic Foreign Body

**DOI:** 10.7759/cureus.62602

**Published:** 2024-06-18

**Authors:** Frank Lin, Nishit Patel, Vesselin Tomov

**Affiliations:** 1 Internal Medicine, St. Luke's Hospital, Bethlehem, USA; 2 Gastroenterology, St. Luke's University Health Network, Bethlehem, USA

**Keywords:** endoscopy, small bowel enterography, swallowed foreign body, duodenal cecal fistula, weight loss

## Abstract

A duodenal-cecal fistula is characterized as an unnatural connection between the duodenum and cecum. Here, we present the case of a 40-year-old male with unintentional weight loss and a history of foreign body ingestion a few years prior. Computerized tomography (CT) small bowel enterography showed a linear soft tissue tract extending from the inferior aspect of the distal duodenum to the cecum. Ultimately, a diagnosis of duodenal-cecal fistula was made following esophagogastroduodenoscopy (EGD) revealing a fistula in the third part of the duodenum. A duodenal-cecal fistula secondary to foreign body ingestion is rare, with surgical intervention or endoscopic fibrin glue closure being potential treatment modalities if the fistula fails to close spontaneously. Duodenal-cecal fistulas are generally seen secondary to malignancies of the duodenum or colon, peptic ulcers, or inflammatory bowel disease. However, a duodenal-cecal fistula due to a foreign body is rare, thus highlighting the importance of keeping a broad differential, as appropriate in the clinical context.

## Introduction

A duodenal-cecal fistula is an abnormal connection between the duodenum and cecum that occurs rarely, with prevalence in middle age [1]. Such fistulas can form due to benign or malignant etiology such as a tumor that arises from the duodenum or colon. One report estimated the incidence of duodenocolic fistula in the United States to be 1 in 900 colorectal carcinomas [2]. Other cases can be benign such as peptic ulcers, duodenal diverticula, inflammatory bowel disease (IBD), and, more rarely, foreign body ingestion. Benign duodenal-cecal fistulas due to alternative etiologies are particularly rare, as most reported cases involve Crohn’s disease or malignant invasion from advanced colon cancer [3]. We present a unique case of unintentional weight loss due to a duodenal-cecal fistula caused by ingestion of a chronic foreign body.

This article was previously presented as a meeting abstract at the 2023 ACG Annual Scientific Meeting on October 22, 2023 [4].

## Case presentation

**Figure 1:**
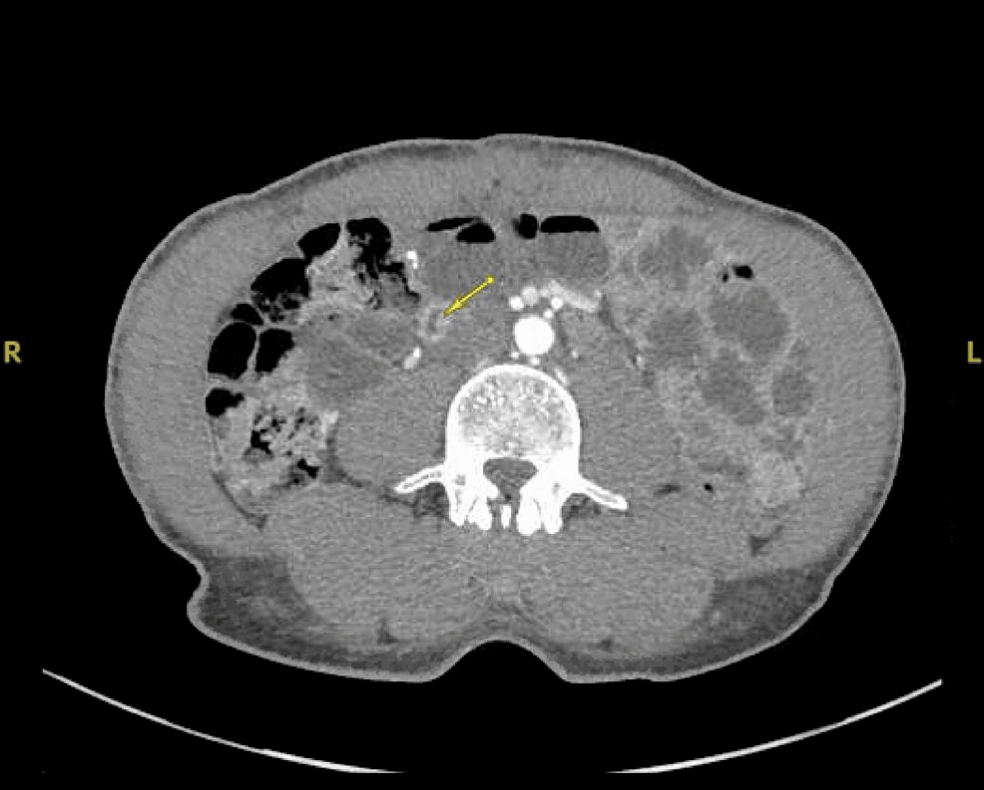
CT small bowel enterography (cross-sectional view) showing soft tissue tract extending from the distal duodenum to the cecum

**Figure 2:**
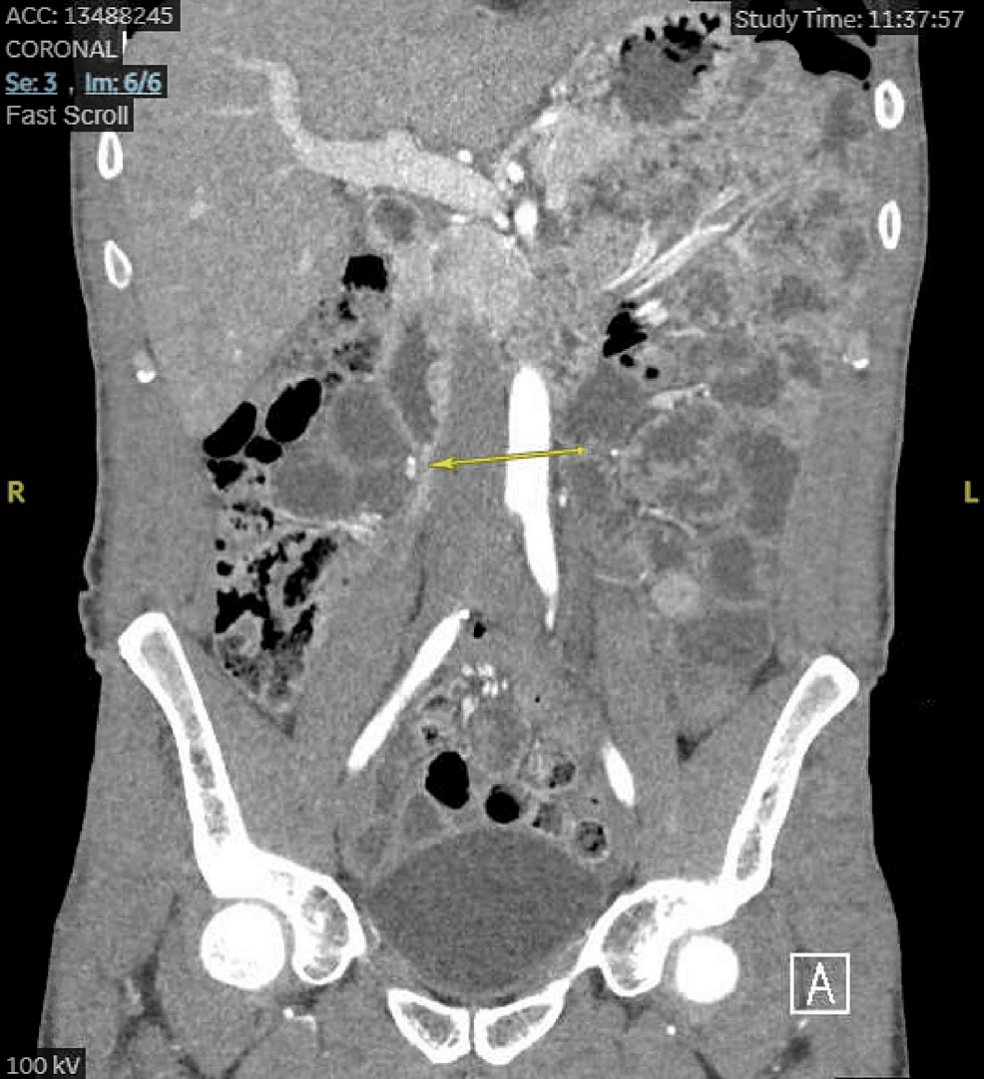
CT small bowel enterography (coronal view) showing soft tissue extending from the distal duodenum to the cecum

**Figure 3:**
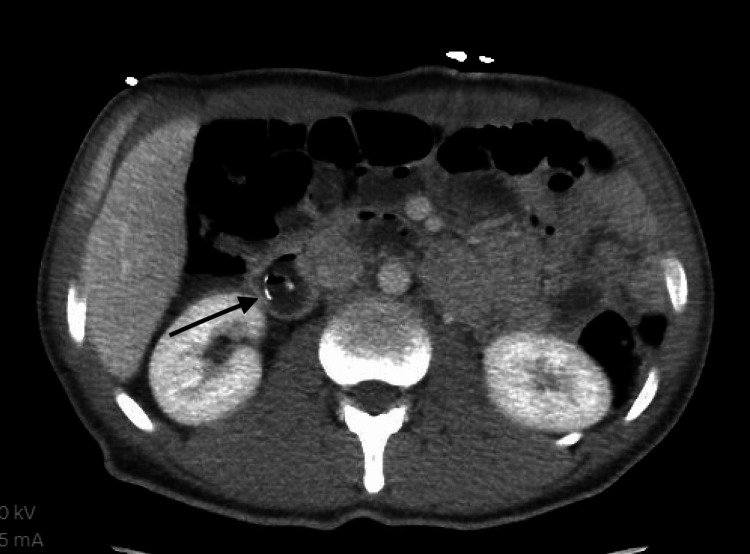
CT abdomen with contrast obtained six years prior with a finding of an unusual radiopaque tubular foreign body extending from the duodenum vertically into the colon

**Figure 4:**
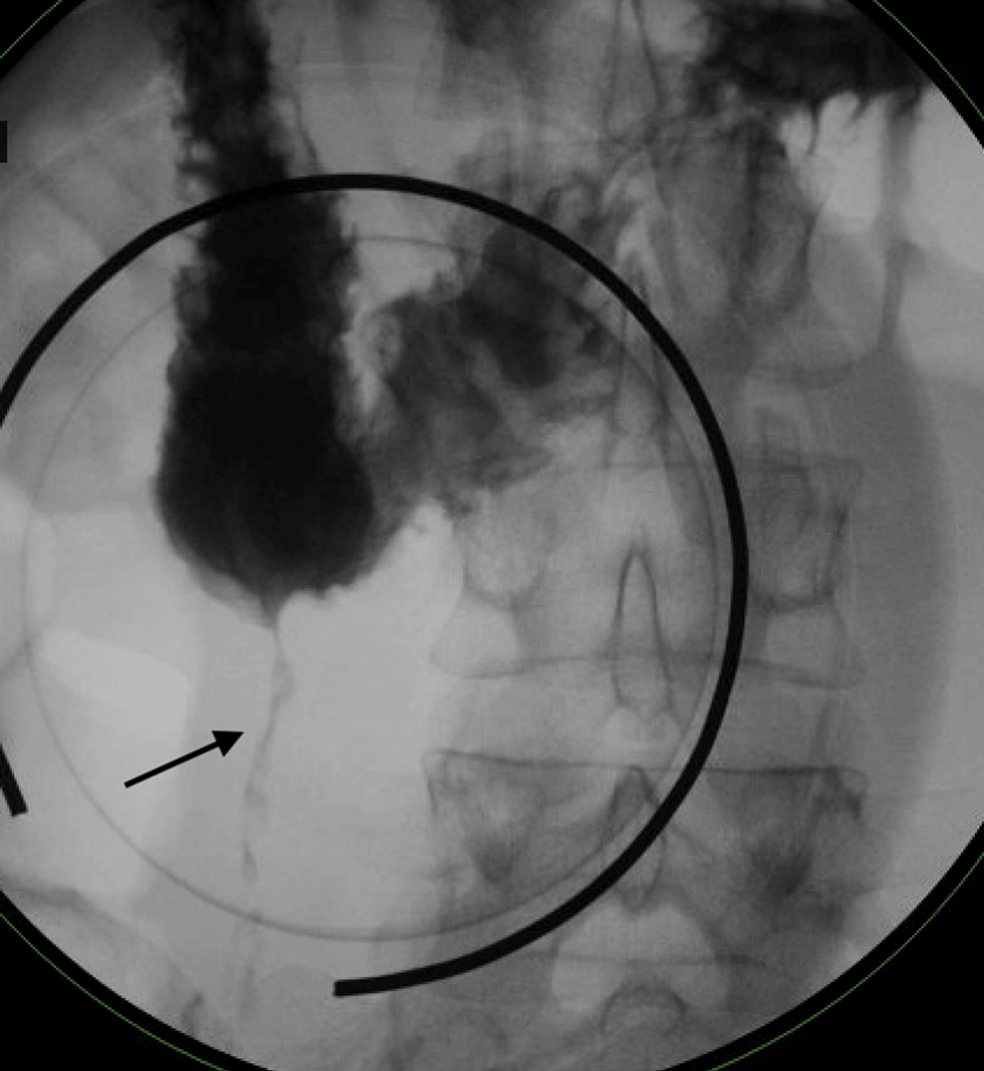
Upper gastrointestinal series obtained six years prior showing a 7-cm-long fistulous communication between the duodenum (at the junction of its second and third portions) and the cecum without barium extravasation

A 40-year-old male presented for unintentional weight loss for two months, with nausea, vomiting, abdominal pain, and early satiety. History was significant for multiple small bowel obstructions and abdominal surgeries, ventral hernia repair, and recurrent foreign body ingestion in the form of a plastic fork presumed secondary to undiagnosed psychiatric illness. A comprehensive metabolic panel (CMP) and complete blood count (CBC) obtained a month ago were unremarkable beyond a mildly elevated aspartate aminotransferase (AST) of 69 U/L. Physical exam was only remarkable for an underweight male with generalized abdominal tenderness. Computerized tomography (CT) abdomen and pelvis with contrast showed no evidence of small or large bowel obstruction. A CT small bowel enterography showed a linear soft tissue tract extending from the inferior aspect of the distal duodenum to the cecum (Figures 1, 2). A diagnosis of duodenal-cecal fistula secondary to foreign body ingestion was suspected six years prior with the patient similarly presenting for unintentional weight loss and CT abdomen revealing an unusual tubular radiopaque foreign body extending from the duodenum vertically into the colon (Figure 3). Following the initial CT scan, esophagogastroduodenoscopy (EGD) and colonoscopy were performed revealing a freely mobile foreign object transecting the duodenal lumen and cecal wall identified as a fork and removed successfully. An upper gastrointestinal series revealed a 7-cm-long fistulous communication between the duodenum and the cecum without barium extravasation (Figure 4).

**Figure 5:**
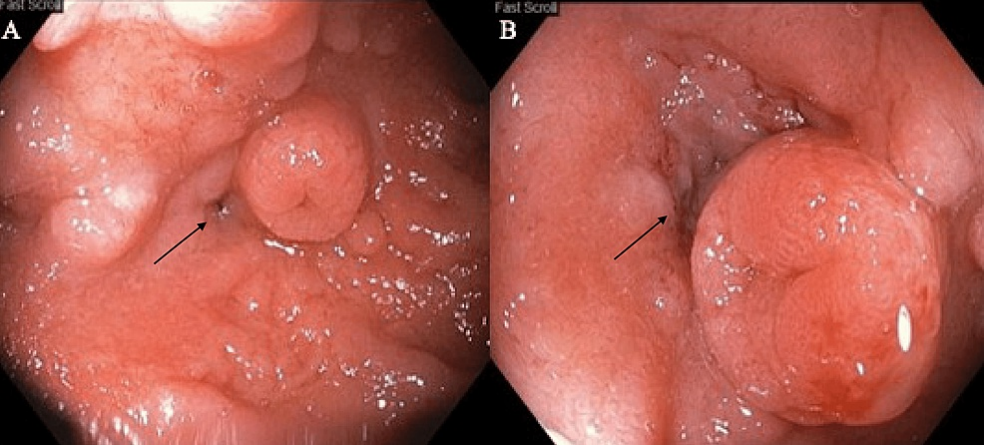
Images A and B obtained during upper endoscopy with visualization of the fistula in the third part of the duodenum

Six years later, a repeat EGD was performed showing persistence of fistula in the third part of the duodenum (Figure 5) noted to be adjacent to the ampulla along with erythematous and nodular mucosa of the stomach and duodenum. Gastric and duodenal biopsies were negative for infection. He was discharged from the hospital with instructions to take a proton pump inhibitor and sucralfate for symptomatic management of nausea and vomiting.

**Figure 6:**
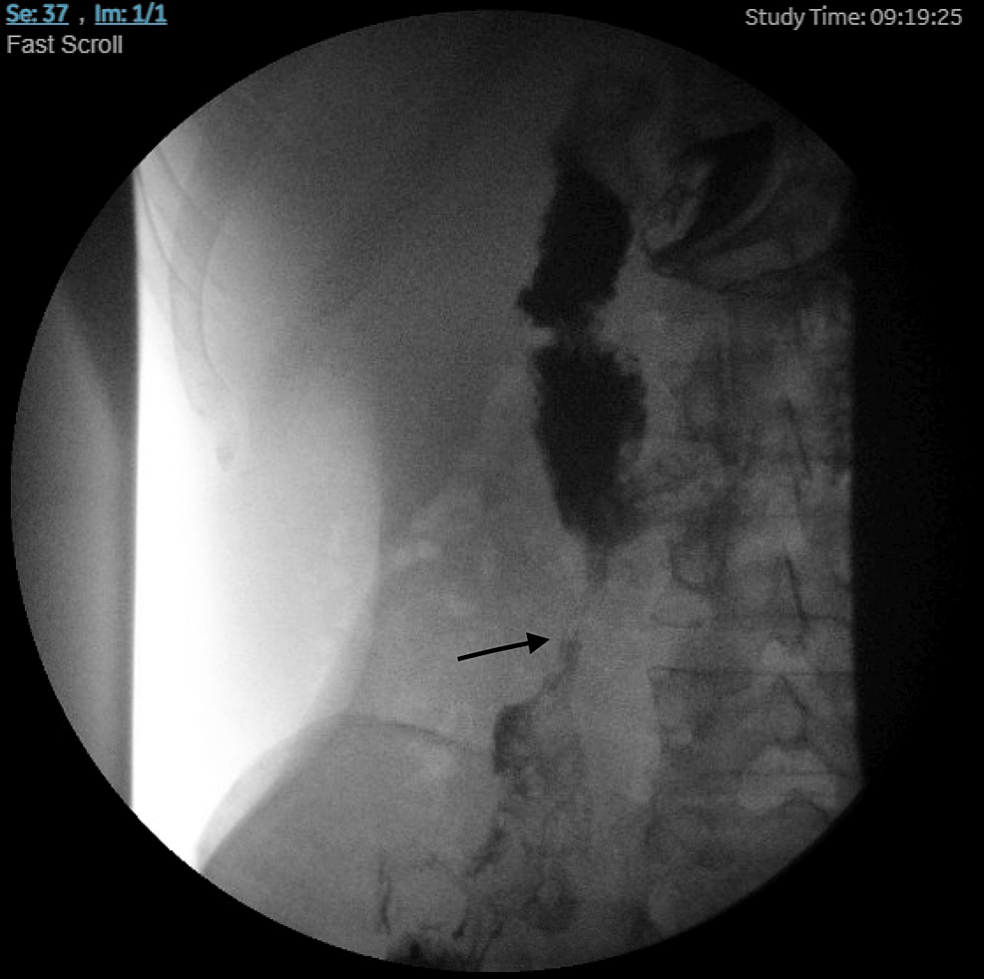
The upper gastrointestinal series reveals contrast flow through a fistulous tract between the second and third portions of the duodenum and cecum

The patient was seen for follow-up as an outpatient and obtained an upper gastrointestinal series, which again showed a fistulous tract between the second and third portions of the duodenum and the cecum (Figure 6). He subsequently underwent repeat EGD a few weeks later for closure of the duodenal-cecal fistula with an endoscopic clip. A year later, following the fistula closure, a repeat upper GI series revealed a residual tract at the junction of the second and third portions of the duodenum without communication with the cecum.

## Discussion

Fistulas of the duodenum and colon are often due to malignancy or inflammatory bowel disease, with a few reported cases of colo-duodenal fistula associated with advanced carcinomas of the hepatic flexure or proximal transverse colon [3]. There has also been a reported case of advanced malignancy of the cecum creating a cecal-duodenal fistula [5]. There are few reported cases of benign duodenal-cecal fistulas, particularly in a patient without a history of malignancy or inflammatory bowel disease. Our patient was seen in the outpatient setting for unintentional weight loss associated with frequent vomiting with suspicion of a duodenal-cecal fistula as the etiology based on imaging findings and known ingestion of a chronic foreign body in the form of a fork that was thought to have passed spontaneously.

Benign causes of duodenal-cecal fistulas include peptic ulcer disease in particular perforated duodenal ulcers [6], Crohn’s disease [7], duodenal diverticulum, tuberculosis, acute cholecystitis, pancreatitis, complication post gastrectomy, and, less commonly, a swallowed foreign body. There have been reported causes of perforated duodenal ulcers resulting in the formation of duodenocolic fistulas [6,8]. Symptoms can typically arise from malignancy or from the fistula itself, with symptoms of abdominal pain, nausea, vomiting, diarrhea, and significant weight loss [9]. The diagnosis of intestinal fistulas requires imaging, and barium enemas can be utilized as a modality to detect duodenal-cecal fistulas due to their ability to visualize fistulous tracts and retrograde flow of the barium [10]. EGD and colonoscopy can allow for direct diagnosis of the fistula and determination of the primary disease. Physicians should have a high index of suspicion for fistula in a patient with a history of foreign body ingestion, with imaging and labs ruling out other causes, including malignancy, inflammatory bowel disease, and small and large bowel obstruction.

Confirmation of the diagnosis of fistula and determination of location, severity, acuity, size, and complexity should guide treatment and management with the primary objective of fistula closure [10]. Some fistulas can close spontaneously but can also be endoscopically closed with fibrin glue and clips or be treated with surgical resection, although the reported success rate of non-operative measures, such as fibrin glue, is low [10]. If obstruction or severe adhesions are present, pursuing right hemicolectomy may be the preferred treatment option [11,12]. The method of treatment should be tailored on a case-by-case basis.

## Conclusions

This case underscores the importance of performing a thorough workup for unintentional weight loss and the need for clinicians to keep duodenal-cecal fistula as a differential, particularly for patients with suggestive imaging findings. Other pathologies, including small or large bowel obstruction due to obstructive malignancy, IBD, or peptic ulcer disease with perforation should be ruled out as possible etiologies for duodenal-cecal fistula. A duodenal-cecal fistula due to foreign body ingestion is particularly rare, emphasizing the need for clinicians to maintain a broad differential.
